# Impregnation of amine functionalized deep eutectic solvents in NH_2_-MIL-53(Al) MOF for CO_2_/N_2_ separation

**DOI:** 10.1038/s41598-023-40191-9

**Published:** 2023-08-10

**Authors:** Narmin Noorani, Abbas Mehrdad

**Affiliations:** Department of Physical Chemistry, Faculty of Chemistry, University of Tabriz, Tabriz, Iran

**Keywords:** Carbon capture and storage, Metal-organic frameworks

## Abstract

To improve the CO_2_/N_2_ separation performance of metal–organic frameworks (MOFs), amine functionalized deep eutectic solvents (DESs) (choline chloride/ethanolamine (DES1), choline chloride/ethanolamine/diethanolamine (DES2), and choline chloride/ethanolamine/methyldiethanolamine (DES3)) confined in the NH_2_-MIL-53(Al). NH_2_-MIL-53(Al) impregnated with DES was synthesized and characterized using N_2_-sorption analysis and Fourier transform infrared (FTIR) spectroscopy. Morphology of the synthesized MOFs was investigated using scanning electron microscopy (SEM). Also, elemental analysis was determined by energy-dispersive X-ray spectroscopy (EDX). CO_2_ adsorption isotherms of amine-functionalized DESs impregnated NH_2_-MIL-53(Al) were measured at temperatures range of 288.15–308.15 K and pressures up to 5 bar. The results reveal that the impregnated MOF with functional group of amine DES improves separation performance NH_2_-MIL-53(Al). CO_2_ adsorption capacity of DES1/NH_2_-MILS-53(Al) was twofold respect to of pristine NH_2_-MIL-53(Al) at 5 bar and 298.15 K; which helps to guide the logical design of new mixtures for gas separation applications. Also, the heat of adsorption for the synthesized NH_2_-MIL-53(Al) and DESs/NH_2_-MIL-53(Al) were estimated. Most importantly, CO_2_ chemisorption by NH_2_ group in the sorbent structure has a significant effect on the adsorption mechanism.

## Introduction

One of the most significant challenges of world is climate change which its prevention and modification received widespread attention. Rapid economic growth and ongoing industrial development have resulted in an increase in the atmospheric carbon dioxide (CO_2_) concentration from 270 ppm before the industrial revolution to 400 ppm now^1,2^. Hence, the emission of CO_2_ has received widespread concern own to its ecosystem changes and environmental effects^3^. Rising global temperatures about 2 °C by the end of this century pose an urgent threat to the planet. The temperature increase of the earth is attributed to the greenhouse gases, of which CO_2_ accounts for more than 70% of the total^4^. To overcome these troubles, the carbon capture and storage concept (CCS) was offered to control the CO_2_ amount in the atmosphere^5^. As a key step in CCS, CO_2_ is required to be captured from CO_2_ emissions.

Technologies to separate CO_2_/N_2_ include membrane separation, molecular sieves, cryogenic separation, and absorption^6–9^. Current technologies employ aqueous amine solutions (monoethanolamine, diethanolamine and methyldiethanolamine), which adsorb CO_2_ at ambient temperature in a thermally reversible manner^10^. Though this adsorption system has numerous advantages such as high reactivity, low cost and good adsorption capacity, it presents some serious disadvantages including solvent loss, emission of VOCs, corrosion and high energy required for the stripping of CO_2_^11^.

To overcome these disadvantages new technologies must be developed. Among the above-mentioned technologies, molecular sieves are of high interest due to the differences in the molecular size, diffusion, and gases affinity. Adsorption and separation of CO_2_ using porous, solid adsorbents as an alternative for amine-based absorption/stripping processes has received much attention during the past decade. Metal–organic frameworks (MOFs), Zeolites, mesoporous silicas, and active carbons adsorbents have been tested for their CO_2_ adsorption behavior^12,13^.

Grafting of amines onto surfaces of porous materials to enhance adsorption of the acidic CO_2_ molecule is another strategy that has been applied for silica-based sorbents and zeolites^14,15^. Arstad et al.^16^ reported CO_2_ adsorption isotherms on three new types of amine-functionalized MOFs. Adsorption capacities of up to 60 wt % were obtained. Dautzenberg et al.^17^ have studied aromatic amine-functionalized covalent organic frameworks (COFs) for CO_2_/N_2_ separation, The COF shows a high CO_2_/N_2_ IAST selectivity under flue gas conditions (273 K: 83 ± 11, 295 K: 47 ± 11). The interaction of the aromatic amine groups with CO_2_ is based on physisorption, which is expected to make the regeneration of the material energy efficient. Ariyanto et al.^18^ have studied DES-impregnated porous carbon which derived from the palm kernel shell for the separation of CO_2_/CH_4_. They indicated that separation performance increased in comparison to pristine porous carbon. Lin et al.^19^ have investigated CO_2_ capture in DES (choline chloride + ethylene glycol) confined into the graphene oxide (GO) with different HBA/HBD molar ratios by molecular dynamics simulation method and concluded that GO provides nanoconfined space for the DES. However, the isotherm data for DESs impregnated on MOFs for separation process is scarce. Metal–organic frameworks (MOFs) are a category of porous materials, which their structure is composed of metal ions networks or metal ion clusters and organic linkers connected through coordination bonds. MOFs own a high internal surface area, tunable multifunctional pores, adaptable porosity, and high thermal and chemical stabilities problems^20,21^. However, the capacity of adsorption of CO_2_ in MOFs is quite low, and hence an enhancement is required. To improve the CO_2_ adsorption capacity, the modification of MOFs using amines particularly monoethanolamine (MEA) can be performed because of availability, low cost, low viscosity, and high affinity to CO_2_^22,23^. However volatile and corrosive nature are disadvantages of amines which led to an unsavory process. Thus, researchers have offered more efficient sorbents such as ionic liquids (ILs) and deep eutectic solvents (DESs) as potential alternatives for conventional amine solutions^24,25^. DESs due to unique properties including high solvation capacity, relatively low cost, higher biodegradability, and nontoxic make them environmentally and technologically superior alternatives to highly expensive ionic liquid^26^. In this research, three-component DESs were prepared by choline chloride:ethanolamine (1:7) (DES1), choline chloride: ethanolamine:diethanolamine (1:7:1) (DES2) and choline chloride:ethanolamine:methyldiethanolamine (1:7:1) (DES3). NH_2_-MIL-53(Al) is composed of octahedral AlO_4_(OH)_2_ linked by a free-standing amine group^27^. In addition, NH_2_-MIL-53(Al) has high pore volume, surface area, and thermal stability^28^. Also, the terephthalate ligands in NH_2_-MIL-53(Al) can increase their compatibility with the DESs. In order to, DESs-impregnated NH_2_-MIL-53(Al) was synthesized for CO_2_ adsorption. The CO_2_ adsorption in these DESs-impregnated NH_2_-MIL-53(Al) was measured using quartz crystal microbalance (QCM). To study the potential of the material for separation purposes, adsorption isotherms were investigated. A novel hybrid model has been offered for correlating CO_2_ isotherm. In addition, CO_2_/N_2_ selectivity was carried out to examine the practical efficacy of the prepared adsorbents.

## Experimental

### Materials

Aluminum nitrate nonahydrate (Al(NO_3_)_3_·9H_2_O) (> 99% purity), 2-amino terephthalic acid (NH_2_-H_2_BDC ≥ 99%), Choline chloride (> 99% purity), Ethanolamine (EA) (≥ 98% purity), diethanolamine (DEA)(≥ 98% purity), and methyldiethanolamine (MDE) (≥ 99% purity) were purchased from Sigma–Aldrich products. *N*,*N*-Dimethylformamide (DMF) (99% purity), Ethanol (> 99% purity) were supplied by Merck. CO_2_ gas (> 99.9% purity) was used in gas absorption tests.

### Synthesis of the DES

In this study, DES based on choline chloride as the HBA and ethanolamine, ethanolamine/diethanolamine, and ethanolamine/methyldiethanolamine as the HBD with special ratio were mixed to determine mole ratios under stirring at 360 K for about 2 h. Then obtained homogeneous solution had melting points below room temperature. The mole ratio of (HBA: HBD) and the melting point of DESs prepared are tabulated in Table 1 which resulted in good agreement with the literature^29^.

**Table 1:** Composition and molar ratio of investigated DESs.

HBA	HBD	DES	Mole ratio	Melting point (K)
Choline chloride	Ethanolamine	DES1	(1:7)	275.2
Choline chloride	Ethanolamine/Diethanolamine	DES2	(1:7:1)	267.0
Choline chloride	Ethanolamine/Methyldiethanolamine	DES3	(1:7:1)	267.8

### Synthesis of NH_2_-MIL-53(Al) and DES-confinement in NH_2_-MIL-53(Al)

Hydrothermal technique was used to synthesis NH_2_-MIL-53(Al). for this purpose, 6.71 g of aluminum nitrate (Al(NO_3_)_3_·9H_2_O) was mixed with 3.74 g of 2-aminoterephthalic acid (NH_2_-H_2_BDC) and 50 mL of deionized water in a Teflon-lined autoclave at 423 K for 5 h. The obtained product was washed with acetone several times. The synthesized NH_2_-MIL-53(Al) was treated at 423 K for about 48 h in DMF to eliminate the unreacted and trapped NH_2_-H_2_BDC in the pores^30^. DESs solution in ethanol with a ratio of 1:1 wt/vol was impregnated in NH_2_-MIL-53(Al) using a vacuum impregnation method. To removal of ethanol, the obtained slurry was dried in oven at 378 K for 24 h.

### Characterization of MOF

A FT-IR (Bruker, Tensor 27) spectrometer was used to recording the FT-IR spectra of NH_2_-MIL-53(Al) and DESs/NH_2_-MIL-53(Al). The morphology of synthesized MOF was investigated using providing FESEM images (EDX & Map & Line). X-ray diffraction (XRD) analysis (SHIMADZU, Labs XRD-6100) was conducted to determine particle size and crystalline structure. EDX spectra was recorded at 10 keV to distinguish the Al of the MOF. Nitrogen adsorption/desorption isotherms were recorded at 77 K (BELSORP MINI II instrument). The samples were degassed at 120 °C under vacuum condition for 10 h prior to the measurements. The BET surface area of the NH_2_-MIL-53(Al) and DESs/NH_2_-MIL-53(Al) were obtained by measuring the nitrogen adsorption at 77 K.

### Gas adsorption apparatus

Gas adsorption measurements were done by QCM sensor. Detail of adsorption apparatus performance has been explained in the prior papers by authors^31–35^. The adsorption capacity, $Q_{e}$ $\left( {{\text{mg}}_{{{\text{CO}}_{{2}} }} \cdot {\text{g}}_{{\text{DESs/MOF}}}^{{ - {1}}} } \right)$ was calculated as follows:

1 $$Q_{e} = \frac{{\Delta F_{S} }}{{\Delta F_{C} }} \times 1000$$

where $\Delta F_{C}$ frequencies difference between the coated crystal with adsorbent and the uncoated crystal. $\Delta F_{S}$ is the difference between the frequencies adsorbent coated crystal under vacuum and after CO_2_ adsorption.

### Thermodynamic model

The CO_2_ experimental isotherms achieved on NH_2_-MIL-53(Al) in this study are correlated to the three-parameter Redlich–Peterson (R–P) model as follows^36^:

2 $$Q = Q_{m} \frac{cp}{{1 + cp^{n} }}$$

where *Q* related to the amount of adsorption per mass of adsorbent $\left( {{\text{mg}}_{{{\text{CO}}_{{2}} }} \cdot {\text{g}}_{{\text{DESs/MOF}}}^{{ - {1}}} } \right)$, *p* is gas pressure at equilibrium condition, *n* is the dimensionless adsorbent parameter and its value was considered as *n* = 1 in this study, also *Q*_*m*_ and *c* are parameters of model. Moreover, to correlate the experimental data of CO_2_ solubility in DESs-impregnated on MOF a hybrid law of Henry and Redlich–Peterson (R–P) model are used as below:

3 $$Q = \frac{p}{H} + Q_{m} \frac{cp}{{1 + cp^{n} }}$$

where *H* is the CO_2_ Henry’s law constant.

The adsorption selectivity for CO_2_/N_2_ was calculated as follows:

$$S_{{CO_{2} /N_{2} }} = \frac{{Q_{{CO_{2} }} }}{{Q_{{N_{2} }} }}$$

where $Q_{{CO_{2} }}$ and $Q_{{N_{2} }}$ are adsorbed values of CO_2_ and N_2_, respectively.

## Results and discussion

### Characterization

#### FT-IR spectra

**Figure 1:**
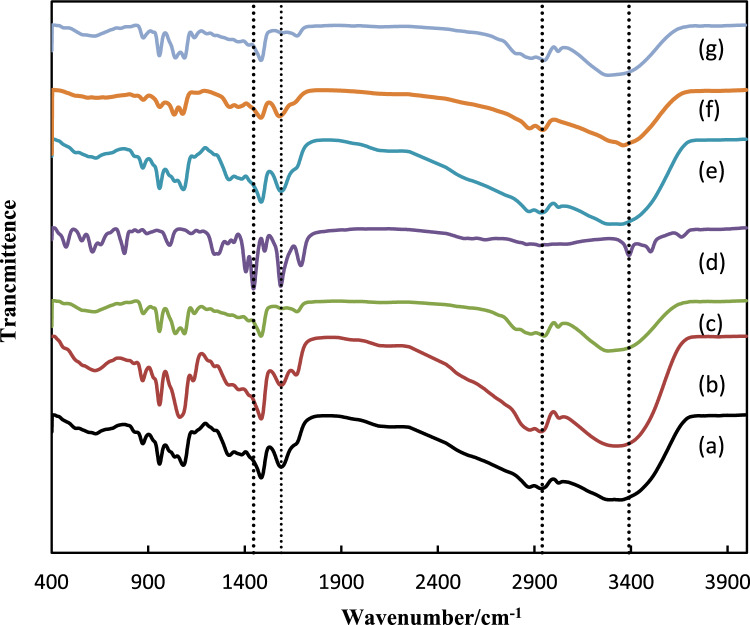
FT-IR spectra of the synthesized materials; (**a**) DES1; (**b**) DES2; (**c**) DES3; (**d**) NH_2_-MIL-53(Al); (**e**) DES1/NH_2_-MIL-53(Al), (**f**) DES2/NH_2_-MIL-53(Al); (**g**) DES3/NH_2_-MIL-53(Al).

The FT-IR spectra of the NH_2_-MIL-53(Al) and DES-impregnated on NH_2_-MIL-53(Al) are illustrated in Fig. 1. As seen from Fig. 1 the symmetric and anti-symmetric N–H and O–H stretching vibrations of DESs are observed at 3300–3500 cm^−1^ and the single C-H stretching vibration is located at 2880–2950 cm^−1^. C–N vibration is observed at 1086 cm^−1^. Also, analysis of FT-IR peaks in Fig. 1d indicates the peak at 3400 cm^−1^ is related to the presence of NH_2_ group MOF. The characteristic peaks at 1612 cm^−1^ and 1402 cm^−1^ are corresponding to the carboxylic acid which coordinated to Al^37^. Also, the peak at 1240 cm^−1^ is attributed to the C–N bending and the band observed at in the range of 965 cm^−1^ is attributed to aromatic C–H in-plane bending. Moreover, another band 629 cm^−1^ correspond with previous observations for MIL-53^38^. The characteristic peaks of both DESs and MOF are observed in DESs/NH_2_-MIL-53(Al) (Fig. 1e–g) which is imply to success impregnation of DESs in MOF.

#### X-ray diffraction (XRD)

**Figure 2:**
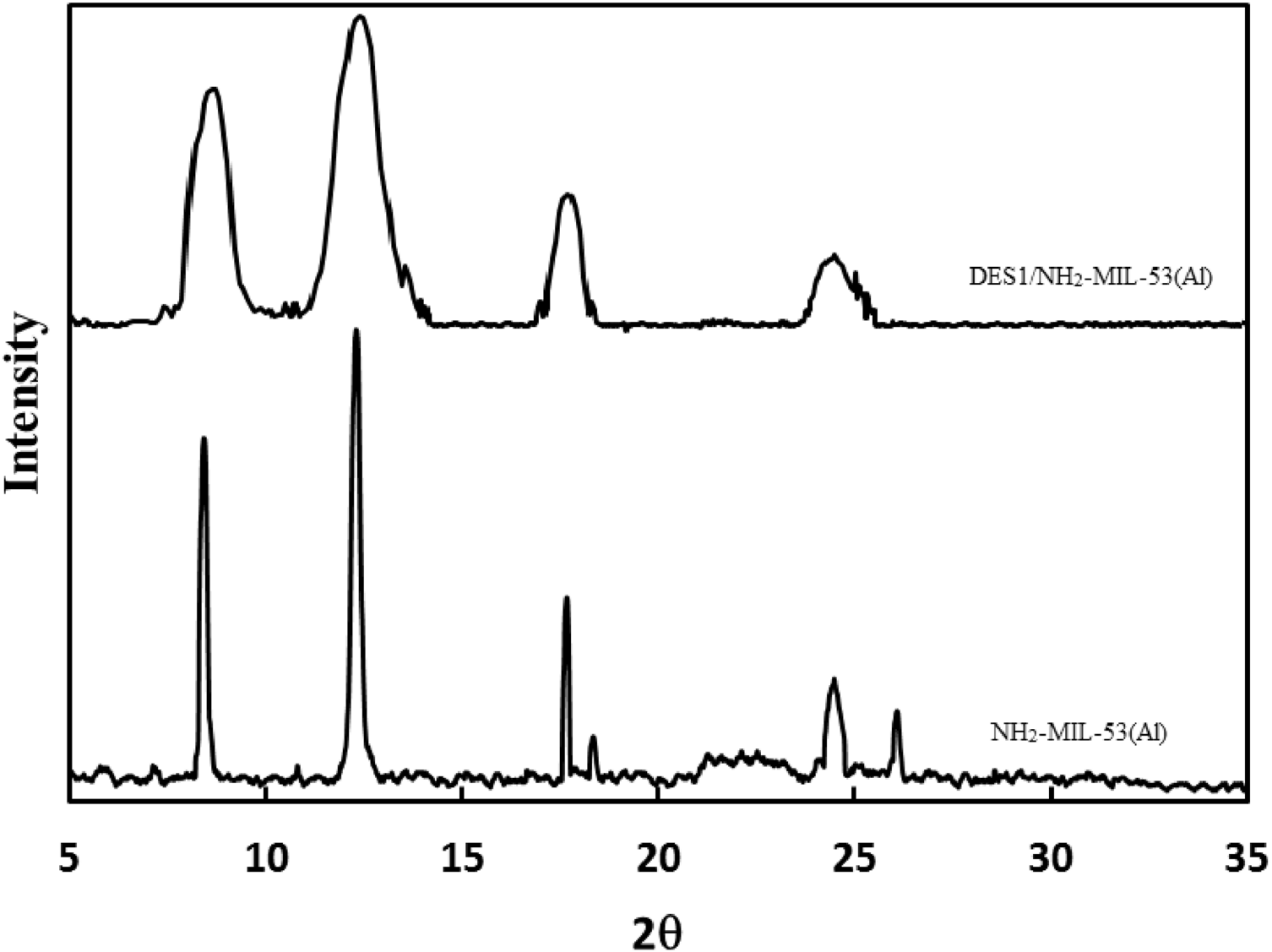
XRD pattern of NH2-MIL-53(Al) and DES1/NH_2_-MIL-53(Al).

The crystalline structure of NH_2_-MIL-53(Al) and DES/NH_2_-MIL-53(Al) were investigated using XRD spectra. XRD pattern of NH2-MIL-53(Al) and DES1/NH_2_-MIL-53(Al) powder are illustrated in Fig. 2. From Fig. 2, it can be observed that the powder sample shows XRD peaks at 2θ values of 8.1°, 12.4°, 17.5°, 24.5° and 25.9° which confirmed the structure of NH2-MIL-53(Al)^39^. The characteristic peaks of NH2-MIL-53(Al) and DES1/NH_2_-MIL-53(Al) are observed at the same angles which imply that crystalline structure of MOF remain unchanged during DES impregnation. However after impregnation the related peaks are broadened which attributed to the decreasing crystallinity degree.

#### EDX pattern

**Figure 3:**
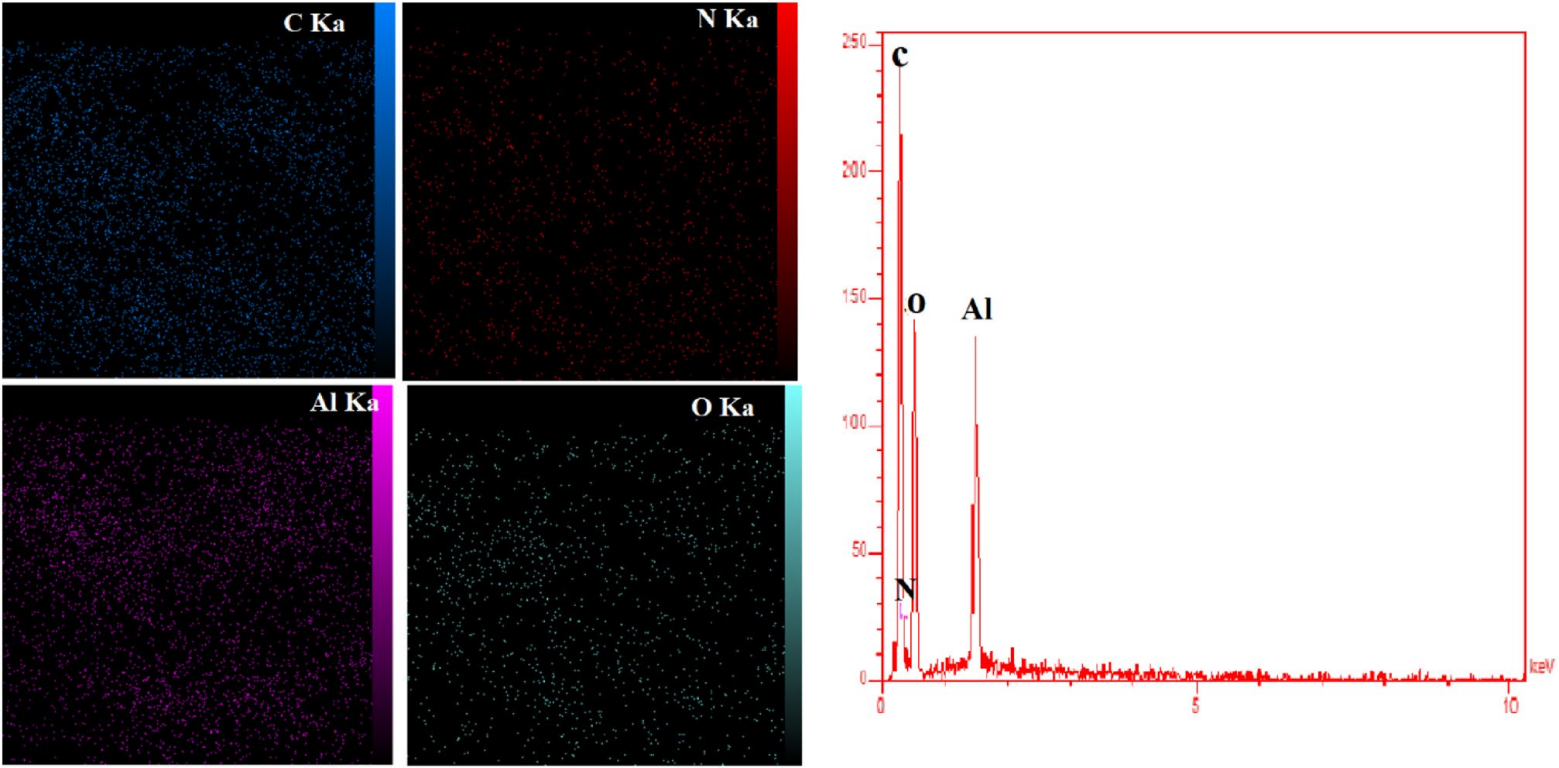
EDX patterns of the synthesized NH_2_-MIL-53(Al).

The distribution of different elements in NH_2_-MIL-53(Al) was identified by EDX analysis. The pattern corresponding to the characteristic elements of NH_2_-MIL-53(Al) is illustrated in Fig. 3. The results of the characteristic elements indicate that the mass fraction of C and N is 56.52% and 8.59%. The corresponding molar ratio of C to N is equal to 6.58, which is close to the molar ratio of C to N in the NH_2_-H_2_BDC structure. These results indicate the purity of the prepared NH_2_-MIL-53 (Al) phase.

#### Scanning electron microscopy

**Figure 4:**
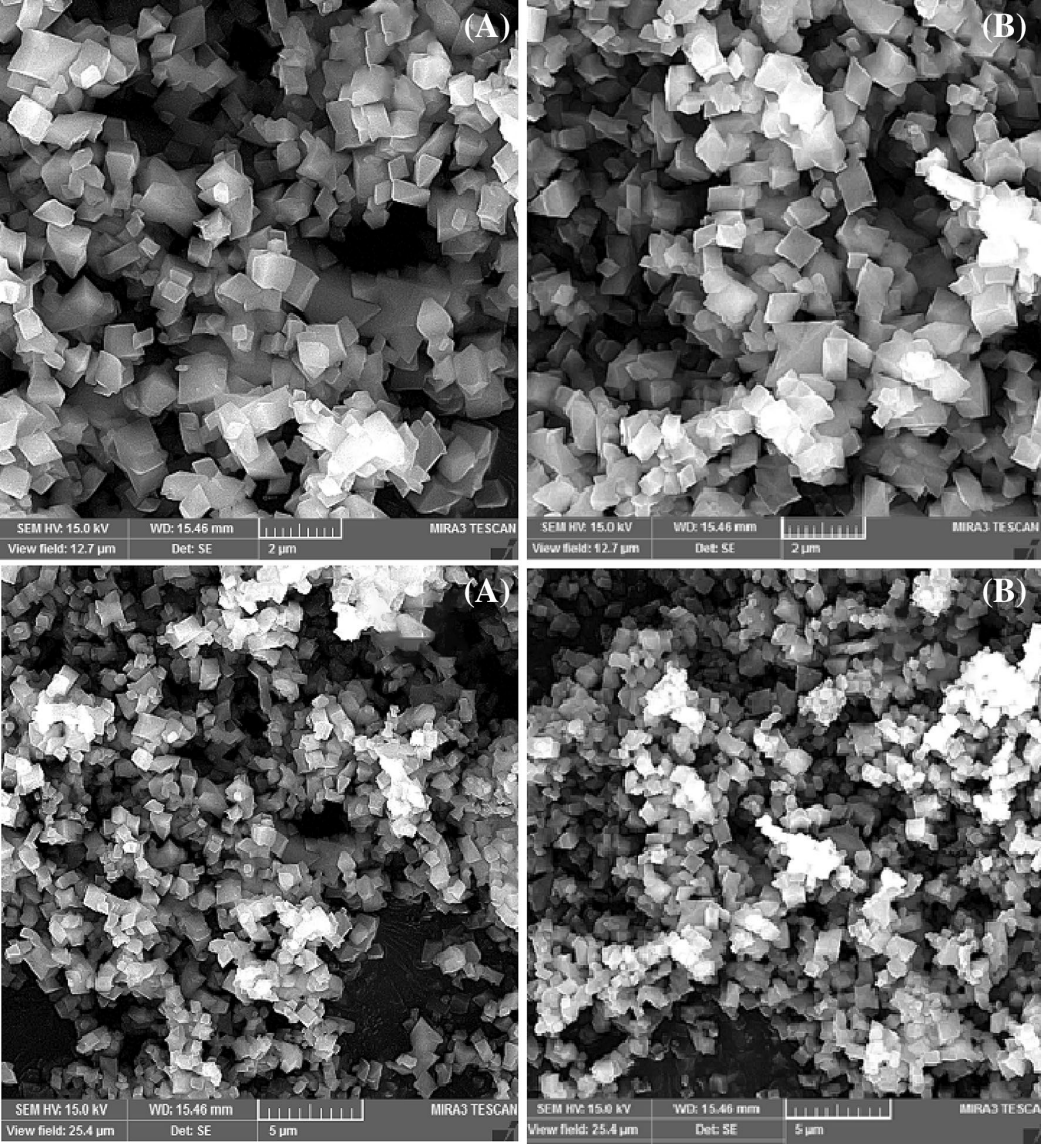
SEM images of (**A**) NH_2_-MIL-53(Al) and (**B**) DES1/NH_2_-MIL-53(Al).

Crystal morphology and size of the products were determined using SEM. SEM image of the NH_2_-MIL-53(Al) is depicted in Fig. 4. As seen in the SEM image, the NH_2_-MIL-53(Al) indicates a three-dimensional hexahedral structure with good regularity.

#### Textural properties of NH_2_-MIL-53(Al)

**Figure 5:**
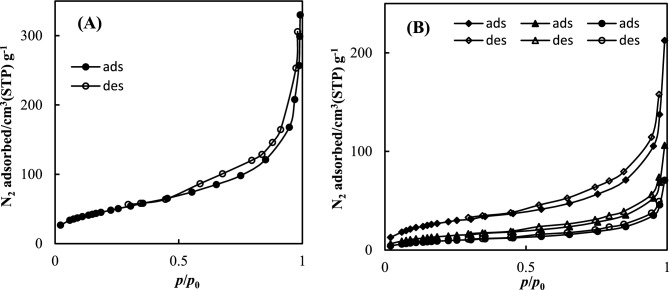
Nitrogen desorption–adsorption isotherms at 77 K: (**A**) in (●) NH_2_-MIL-53(Al); and (**B**) (♦) DES1/NH_2_-MIL-53(Al); (▲) DES2/NH_2_-MIL-53(Al); (●) DES3/NH_2_-MIL-53(Al).

Nitrogen adsorption isotherms at 77 K were used to analyze the samples' textural characteristics, such as their specific surface area (A_BET_), micropore volume (V_MP_), and total pore volume (V_P_). As depicted in Fig. 5, typical of microporous crystalline materials were obtained Type Ib isotherms^40^. In other words, the N_2_ isotherm of NH_2_-MIL-53(Al) showed hysteresis behavior. The NH_2_-MIL-53(Al) and DESs-impregnated NH_2_-MIL-53(Al) samples displayed high volume adsorption at extremely low pressures, virtually achieving the maximum adsorption capacity. According to the isotherm of DES3-impregnated NH_2_-MIL-53(Al), the sample had a low capacity for adsorption and a rise in adsorbed volume at relative pressures above 0.9, which are signs of macropore filling that may be caused by interstitial gaps. The textural properties of NH_2_-MIL-53(Al) and DES/NH_2_-MIL-53(Al) are reported in Table 2. The micropore volume and BET surface area of MOF is comparable with values reported in the literature^28,41^. The textural properties indicated that the impregnation procedure decreased the values of micropore volume, specific surface area and total pore volume relative to the original NH_2_-MIL-53(Al) sample. Moreover, DES3/NH_2_-MIL-53(Al), the higher the reduction in textural parameters and N_2_ adsorbed volume at 77 K. This behavior reveals that DES molecules were incorporated in the pores of NH_2_-MIL-53(Al) because of the impregnation process.

**Table 2:** Textural characteristics of the samples.

DES /NH_2_-MIL-53	A_BET_/m^2^ g^−1^	V_P_/cm^3^ g^−1^	V_MP_/cm^3^ g^−1^
NH_2_-MIL-53(Al)	635	0.32	0.27
DES1/NH_2_-MIL-53(Al)	480	0.29	0.23
DES2/NH_2_-MIL-53(Al)	435	0.25	0.21
DES3/NH_2_-MIL-53(Al)	398	0.22	0.19

### Adsorption isotherms

**Figure 6:**
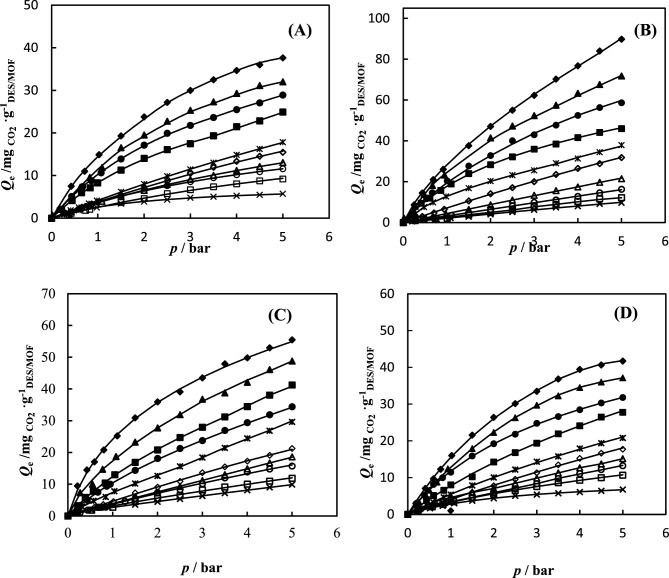
The CO_2_ adsorption in (**A**) NH_2_-MIL-53(Al); (**B**) DES1/NH_2_-MIL-53(Al); (**C**) DES2/NH_2_-MIL-53(Al); (**D**) DES3/NH_2_-MIL-53(Al) at different temperatures (♦) 288.15 K; (▲) 293.15 K; (●) 298.15 K; (■) 303.15 K; (*) 308.15 K; the N_2_ adsorption at different temperatures (◊) 288.15 K; (∆) 293.15 K; (○) 298.15 K; (□) 303.15 K; (✖) 308.15 K; (–) fitting results by Eq. (3).

**Figure 7:**
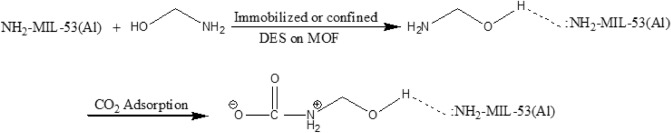
Schematic interaction of DES with MOF and CO_2_.

**Figure 8:**
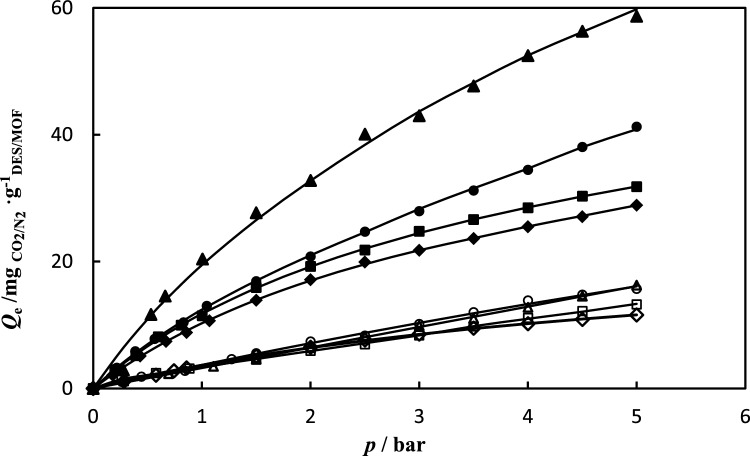
(**A**) The CO_2_ adsorption in (♦) NH_2_-MIL-53(Al); (▲) DES1/NH_2_-MIL-53(Al); (●) DES2/NH_2_-MIL-53(Al); (■) DES3/NH_2_-MIL-53(Al); and N_2_ adsorption in (◊) NH_2_-MIL-53(Al); (∆) DES1/NH_2_-MIL-53(Al); (○) DES2/NH_2_-MIL-53(Al); (□) DES3/NH_2_-MIL-53(Al); at temperatures 298.15 K; (–) fitting results by Eq. (3).

**Figure 9:**
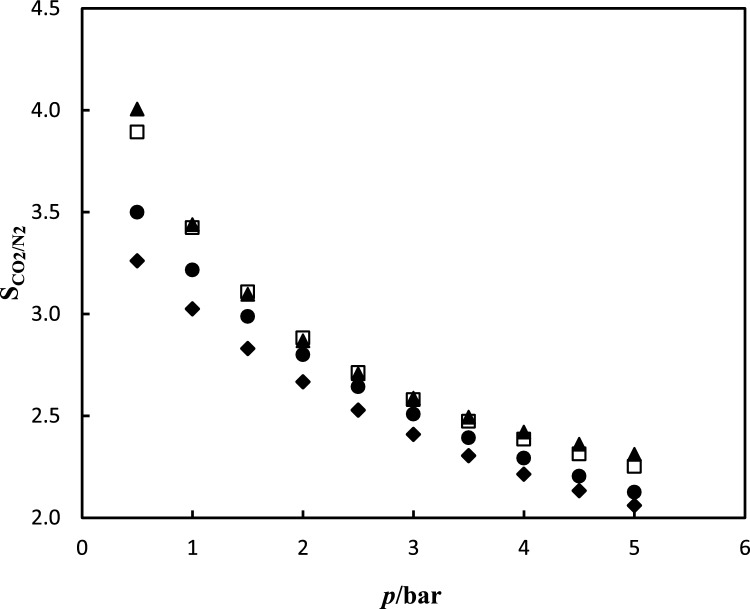
Selectivity of CO_2_/N_2_ versus pressure at temperature 293.15 K; (♦) NH_2_-MIL-53(Al); (▲) DES1/NH_2_-MIL-53(Al); (●) DES2/NH_2_-MIL-53(Al); (□) DES3/NH_2_-MIL-53(Al).

CO_2_ and N_2_ gas adsorption in NH_2_-MIL-53(Al) and DESs-impregnated NH_2_-MIL-53(Al) were measured at range temperature of 288.15–308.15 K and pressure of up to 5 bar. The CO_2_ and N_2_ gas adsorption data are tabulated in Tables 3 and 4. The experimental data of CO_2_ and N_2_ gas adsorption in NH_2_-MIL-53(Al) and DESs-impregnated NH_2_-MIL-53(Al) is correlated by the hybrid model. The parameters of the Redlich–Peterson (R–P) model $q_{m}$ and *c*, and Henry’s constant (*H*), for CO_2_ and N_2_ on the synthesized NH_2_-MIL-53(Al) and DESs/NH_2_-MIL-53(Al) are reported in Tables 5 and 6, respectively. The absolute average relative deviation is lower than 0.02, which implies to suitable capability of the proposed model. The calculated data indicate that the amount of CO_2_ adsorbed was more than the amount of N_2_ adsorbed. According to the data, the DESs-impregnated NH_2_-MIL-53(Al) exhibit stronger adsorption and increases the CO_2_ adsorption capacity. NH_2_-MIL-53(Al) has a CO_2_ adsorption capacity of 28.87 ${\text{mg}}_{{{\text{CO}}_{{2}} }} \cdot {\text{g}}_{{{\text{NH}}_{{2}} {\text{ - MIL - 53}}}}^{{ - {1}}}$, while DESs-impregnated NH_2_-MIL-53(Al), DES1/NH_2_-MIL-53(Al), DES2/NH_2_-MIL-53(Al), and DES3/NH_2_-MIL-53(Al) have adsorption capacities of 62.68, 39.66, and 31.84 ${\text{mg}}_{{{\text{CO}}_{{2}} }} \cdot {\text{g}}_{{{\text{DES/NH}}_{{2}} {\text{ - MIL - 53}}}}^{{ - {1}}}$ , respectively at temperature of 298.15 K and pressure 5 bar. The isotherm curves of CO_2_ and N_2_ gas adsorption of the studied systems at different temperatures and pressures are illustrated in Fig. 6. In NH_2_-MIL-53(Al) main interaction between CO_2_ and MOF arises from carboxylate oxygen atoms and –NH_2_ group in MOF, can increase preferential interactions between the frameworks and CO_2_. In the MOF impregnated with DESs, gas adsorption arises from two factor; first factor is DESs which confinement in pores and second factor is DESs which immobilized in the surface of pores. The schematic interaction of DES with NH_2_-MIL-53(Al) and CO_2_ are illustrated in Fig. 7. With impregnation of DES in MOF a monolayer of DES is immobilized on the surface of MOF pores via hydrogen bond interactions. However residual DES so far away from surface which could not forming hydrogen bond. Therefore except of monolayer, the residual of DES is confined in the pores of MOF. When most of the pore surface of support is occupied by the immobilized DESs, the CO_2_ sorption capacity of nano-confined DESs is dominated by the immobilized DESs rather than solid adsorbent. In the NH_2_-MIL-53(Al) impregnated with DESs, at low pressure gas adsorption take placed on the immobilized DESs on surface of NH_2_-MIL-53(Al); whereas at high pressure gas adsorption take placed on the confinement DESs on pores of NH_2_-MIL-53(Al). Moreover, the chemical reaction between CO_2_ molecules and the amine group affects the CO_2_ adsorption NH_2_-MIL-53(Al). Improving the pore characteristics of NH_2_-MIL-53(Al) with different active sites, among which the creation of carboxylate oxygen atoms and NH_2_ functional group, can increase preferential interactions between the frameworks and CO_2_. The comparison of adsorption isotherm in DESs-impregnated NH_2_-MIL-53(Al) was illustrated in Fig. 8 at temperature of 298.15 K and pressure of up to 5 bar. The values of adsorption capacity follow the trend of: DES1/NH_2_-MIL-53(Al) > DES3/NH_2_-MIL-53(Al) > DES2/NH_2_-MIL-53(Al). The highest CO_2_ adsorption is corresponding to DES1/NH_2_-MIL-53(Al) and the lowest adsorption is attributed to DES2/NH_2_-MIL-53(Al), indeed, the addition of the secondary amine to DES1/NH_2_-MIL-53(Al) reduced CO_2_ adsorption. Reactivity of amines with CO_2_ is in the order as Primary > Secondary > Tertiary. Hence, the addition of secondary amine decreased the absorption capacity of primary amine. Also, in DES3/NH_2_-MIL-53(Al), when the tertiary amine is added to the DES1/NH_2_-MIL-53(Al) adsorption capacity decreases however in compared to the DES2/NH_2_-MIL-53(Al) the adsorption capacity increased due to the stronger intermolecular hydrogen bonds in DEA, consequently reaction between MDE and CO_2_ is easier than reaction between DEA and CO_2_ which leads to increase values of adsorption capacity. Overall, impregnation of MOF with DES1, DES2 and DES3 enhance the adsorption capacity about 130%, 14% and 52%, respectively. The similar results were reported in literatures. For example, Ariyanto et al.^18^ have studied adsorption capacity of porous carbon which impregnated with three DESs (choline chloride:butanol, choline chloride:ethylene glycol and choline chloride:glycerol). They results reveals that the enhance adsorption capacity were 68%, 71% and 95%, respectively. The selectivity of CO_2_/N_2_ for NH_2_-MIL-53(Al) and DESs/NH_2_-MIL-53(Al) are shown in Fig. 9. The value of CO_2_/N_2_ selectivity decreases with increase in the pressure which is in good agreement with the literature^42,43^. DES1/NH_2_–MIL–53(Al) displays batter selectivity with respect to the NH_2_-MIL-53(Al).

**Table 3:** CO_2_ adsorption capacity $Q_{e}$ $\left( {{\text{mg}}_{{{\text{CO}}_{{2}} }} \cdot {\text{g}}_{{{\text{DES/NH}}_{{2}} {\text{ - MIL - 53}}}}^{{ - {1}}} } \right)$ of NH_2_-MIL-53(Al), DES1/NH_2_-MIL-53(Al), DES2/NH_2_-MIL-53(Al), and DES3/NH_2_-MIL-53(Al) at 288.15–308.15 K temperature range and pressures up to 5 bar.

*T* = 288.15 K		*T* = 293.15 K		*T* = 298.15 K		*T* = 303.15 K		*T* = 308.15 K	
*p*/bar	$Q_{e}$/${\text{mg}}_{{{\text{CO}}_{{2}} }} {\text{.g}}_{{\text{DES/MOF}}}^{{ - {1}}}$	*p*/bar	$Q_{e}$/${\text{mg}}_{{{\text{CO}}_{{2}} }} {\text{.g}}_{{\text{DES/MOF}}}^{{ - {1}}}$	*p*/bar	$Q_{e}$/${\text{mg}}_{{{\text{CO}}_{{2}} }} {\text{.g}}_{{\text{DES/MOF}}}^{{ - {1}}}$	*p*/bar	$Q_{e}$/${\text{mg}}_{{{\text{CO}}_{{2}} }} {\text{.g}}_{{\text{DES/MOF}}}^{{ - {1}}}$	*p* /bar	$Q_{e}$/${\text{mg}}_{{{\text{CO}}_{{2}} }} {\text{.g}}_{{\text{DES/MOF}}}^{{ - {1}}}$
NH_2_-MIL-53(Al)									
0.422	7.3278	0.017	0.7	0.180	2.085	0.213	2.071	0.220	0.915
0.713	11.247	0.436	4.664	0.433	5.096	0.442	4.143	0.411	1.829
1.025	14.6844	0.615	7.229	0.671	7.413	0.646	5.984	0.590	2.515
1.500	19.121	0.824	9.562	0.859	8.802	0.834	7.135	0.807	3.430
2.000	23.273	1.090	11.894	1.070	10.656	0.995	8.285	0.978	4.116
2.500	27.088	1.500	16.558	1.500	13.899	1.500	11.277	1.500	6.174
3.000	29.971	2.000	19.522	2.000	17.142	2.000	14.039	2.000	8.004
3.500	32.482	2.500	22.754	2.500	19.921	2.500	16.110	2.500	9.833
4.000	34.66	3.000	25.318	3.000	21.774	3.000	17.491	3.000	11.434
4.500	36.503	3.500	27.317	3.500	23.628	3.500	19.333	3.500	13.035
5.000	37.643	4.000	29.282	4.000	25.481	4.000	21.483	4.000	14.864
		4.500	31.147	4.500	27.071	4.500	22.834	4.500	16.236
		5.000	32.013	5.000	28.897	5.000	24.915	5.000	17.837
DES1/NH_2_-MIL-53(Al)									
0.250	8.689	0.017	2.182	0.278	2.915	0.218	4.414	0.477	7.549
0.436	14.482	0.272	8.000	0.532	11.662	0.412	9.458	0.682	9.872
0.685	20.999	0.480	13.818	0.663	14.577	0.610	11.98	0.979	12.776
0.915	26.068	0.668	18.182	1.006	20.408	0.860	15.763	1.500	16.841
1.500	37.654	0.945	23.273	1.500	27.697	1.096	18.916	2.000	20.325
2.000	47.067	2.000	41.455	2.000	32.799	1.500	23.96	2.500	23.229
2.500	55.033	2.500	47.273	2.500	40.087	2.000	28.373	3.000	25.552
3.000	62.274	3.000	52.364	3.000	43.003	2.500	32.156	3.500	29.036
3.500	70.239	3.500	57.455	3.500	47.705	3.000	35.939	4.000	31.359
4.000	76.756	4.000	63.273	4.000	52.478	3.500	38.462	4.500	34.482
4.500	83.997	4.500	67.636	4.500	56.309	4.000	41.614	5.000	37.885
5.000	89.790	5.000	71.773	5.000	58.682	4.500	44.136		
						5.000	46.028		
DES2/NH_2_-MIL-53(Al)									
0.191	2.976	0.267	3.289	0.199	2.959	0.245	2.192	0.245	1.435
0.409	7.068	0.502	6.579	0.392	5.178	0.436	4.019	0.436	2.512
0.599	9.673	0.682	8.772	0.599	8.136	0.601	5.115	0.601	3.229
0.767	12.277	0.933	12.427	0.810	9.985	0.839	6.577	0.839	4.665
1.025	15.997	1.500	17.909	1.003	11.464	1.003	8.038	1.003	5.382
1.500	21.577	2.000	22.295	1.500	15.902	1.500	10.230	1.500	7.894
2.000	26.414	2.500	26.316	2.000	19.231	2.000	14.249	2.000	10.047
2.500	30.134	3.000	29.702	2.500	21.820	2.500	16.807	2.500	12.199
3.000	33.482	3.500	32.260	3.000	24.778	3.000	19.364	3.000	14.352
3.500	36.830	4.000	34.530	3.500	26.627	3.500	21.922	3.500	15.788
4.000	39.435	4.500	36.012	4.000	28.476	4.000	24.114	4.000	17.940
4.500	40.667	5.000	37.170	4.500	30.325	4.500	26.472	4.500	19.376
5.000	41.712			5.000	31.805	5.000	27.768	5.000	20.811
DES3/NH_2_-MIL-53(Al)									
0.213	9.458	0.272	7.353	0.218	3.251	0.267	3.127	0.313	3.007
0.423	14.502	0.546	10.027	0.387	5.852	0.428	5.003	0.569	4.811
0.591	17.024	0.773	14.706	0.566	7.802	0.666	7.505	0.845	6.013
0.799	20.807	1.072	18.717	0.831	10.403	0.863	9.381	1.036	7.817
1.099	25.221	1.500	23.270	1.045	13.004	0.943	10.006	1.500	10.222
1.500	30.895	2.000	27.738	1.500	16.905	1.500	14.384	2.000	12.628
2.000	35.939	2.500	31.917	2.000	20.806	2.000	18.136	2.500	15.634
2.500	39.092	3.000	36.765	2.500	24.707	2.500	21.263	3.000	18.438
3.000	43.506	3.500	38.770	3.000	27.958	3.000	23.765	3.500	21.460
3.500	47.919	4.000	42.112	3.500	31.209	3.500	26.941	4.000	24.452
4.000	49.811	4.500	46.123	4.000	34.460	4.000	29.393	4.500	26.860
4.500	52.963	5.000	48.797	4.500	38.061	4.500	31.895	5.000	29.661
5.000	55.485			5.000	41.262	5.000	34.396		

Standard uncertainties are u ($Q_{e}$) = 0.010, u(*T*) = 0.05 K, and u (*p*) = 0.005.

**Table 4:** N_2_ adsorption capacity $Q_{e}$ (${\text{mg}}_{{{\text{CO}}_{{2}} }} .{\text{g}}_{{{\text{DES/NH}}_{{2}} {\text{ - MIL - 53}}}}^{{ - {1}}}$) of NH_2_-MIL-53(Al), DES1/NH_2_-MIL-53(Al), DES2/NH_2_-MIL-53(Al), and DES3/NH_2_-MIL-53(Al) at 288.15–308.15 K temperature range and pressures up to 5 bar.

*T* = 288.15 K		*T* = 293.15 K		*T* = 298.15 K		*T* = 303.15 K		*T* = 308.15 K	
*p*/bar	$Q_{e}$/${\text{mg}}_{{{\text{CO}}_{{2}} }} .{\text{g}}_{{\text{DES/MOF}}}^{ - 1}$	*p*/bar	$Q_{e}$/${\text{mg}}_{{{\text{CO}}_{{2}} }} .{\text{g}}_{{\text{DES/MOF}}}^{ - 1}$	*p*/bar	$Q_{e}$/${\text{mg}}_{{{\text{CO}}_{{2}} }} .{\text{g}}_{{\text{DES/MOF}}}^{ - 1}$	*p*/bar	$Q_{e}$/${\text{mg}}_{{{\text{CO}}_{{2}} }} .{\text{g}}_{{\text{DES/MOF}}}^{ - 1}$	*p*/bar	$Q_{e}$/${\text{mg}}_{{{\text{CO}}_{{2}} }} \cdot {\text{g}}_{{\text{DES/MOF}}}^{ - 1}$
NH_2_-MIL-53(Al)									
0.302	1.172	0.376	1.399	0.285	1.158	0.376	0.921	0.275	0.915
0.656	2.577	0.615	2.332	0.578	2.085	0.422	1.151	0.421	1.372
0.873	3.280	0.824	3.032	0.743	2.780	0.734	1.841	0.590	1.829
0.987	3.749	1.090	3.965	0.859	3.243	0.825	2.071	0.978	2.515
1.500	5.389	1.500	5.131	1.500	5.096	1.500	3.682	1.500	3.430
2.000	7.029	2.000	6.530	2.000	6.486	2.000	4.603	2.000	3.887
2.500	8.669	2.500	7.929	2.500	7.644	2.500	5.754	2.500	4.345
3.000	10.544	3.000	9.095	3.000	8.571	3.000	6.674	3.000	4.802
3.500	11.949	3.500	10.028	3.500	9.497	3.500	7.365	3.500	5.031
4.000	13.355	4.000	11.194	4.000	10.192	4.000	8.055	4.000	5.260
4.500	14.527	4.500	12.127	4.500	10.887	4.500	8.746	4.500	5.488
5.000	15.464	5.000	13.060	5.000	11.582	5.000	9.206	5.000	5.717
DES1/NH_2_-MIL-53(Al)									
0.185	1.179	0.477	1.742	0.280	1.163	0.272	0.954	0.207	0.572
0.436	2.946	0.682	2.904	0.694	2.327	0.466	1.723	0.486	1.145
0.703	4.714	0.979	4.065	1.107	3.490	0.736	2.084	0.768	1.717
0.940	6.482	1.500	6.388	1.500	4.654	1.008	2.065	1.082	2.290
1.500	10.607	2.000	8.711	2.000	6.399	1.500	3.807	1.500	2.862
2.000	14.143	2.500	11.034	2.500	8.144	2.000	4.969	2.000	4.007
2.500	17.089	3.000	13.357	3.000	9.889	2.500	6.130	2.500	5.152
3.000	20.035	3.500	15.679	3.500	11.053	3.000	8.012	3.000	6.297
3.500	23.571	4.000	17.422	4.000	12.798	3.500	8.872	3.500	7.441
4.000	26.517	4.500	19.744	4.500	14.543	4.000	10.340	4.000	8.014
4.500	28.874	5.000	21.487	5.000	16.289	4.500	11.614	4.500	9.159
5.000	31.821					5.000	12.195	5.000	9.731
DES2/NH_2_-MIL-53(Al)									
0.294	1.063	0.294	1.762	0.261	1.394	0.237	1.033	0.212	0.677
0.638	2.481	0.638	3.524	0.579	2.440	0.571	2.066	0.516	1.693
1.001	4.252	1.001	4.581	0.885	3.137	0.822	2.755	0.813	2.37
1.500	6.024	1.500	5.990	1.500	4.531	1.068	3.444	1.080	3.047
2.000	7.796	2.000	7.047	2.000	5.925	1.500	4.477	1.500	3.724
2.500	9.568	2.500	8.457	2.500	6.971	2.000	5.510	2.000	4.401
3.000	11.339	3.000	9.866	3.000	8.365	2.500	6.543	2.500	5.078
3.500	13.466	3.500	11.276	3.500	9.759	3.000	7.576	3.000	5.416
4.000	15.237	4.000	12.685	4.000	11.105	3.500	8.609	3.500	5.755
4.500	16.655	4.500	13.742	4.500	12.199	4.000	9.298	4.000	6.093
5.000	17.718	5.000	15.152	5.000	13.245	4.500	9.986	4.500	6.432
						5.000	10.675	5.000	6.77
DES3/NH_2_-MIL-53(Al)									
0.166	0.910	0.180	0.930	0.270	1.017	0.213	0.913	0.226	0.903
0.414	1.820	0.632	2.791	0.444	1.848	0.527	1.826	0.535	1.805
0.591	2.730	0.943	3.721	0.845	2.773	0.717	2.740	0.904	2.708
0.788	3.640	1.500	5.581	1.274	4.621	1.001	2.740	1.500	3.610
1.017	4.550	2.000	7.442	1.500	5.545	1.500	4.566	2.000	4.513
1.500	7.279	2.500	9.302	2.000	7.394	2.000	5.479	2.500	5.415
2.000	9.099	3.000	11.163	2.500	8.318	2.500	6.393	3.000	6.318
2.500	10.919	3.500	13.023	3.000	10.166	3.000	8.219	3.500	7.220
3.000	13.649	4.000	14.884	3.500	12.015	3.500	9.132	4.000	8.123
3.500	15.469	4.500	16.744	4.000	13.863	4.000	10.046	4.500	9.025
4.000	17.288	5.000	18.605	4.500	14.787	4.500	10.959	5.000	9.928
4.500	19.108	0.180	0.930	5.000	15.712	5.000	11.872		
5.000	21.258								

Standard uncertainties are u ($Q_{e}$) = 0.010, u(*T*) = 0.05 K, and u (*p*) = 0.005.

**Table 5:** Henry’s law constant (*H)*, $Q_{m}$ and *c* are also parameters of the Redlich–Peterson isotherm model, the correlation coefficient (*R*^2^) and absolute average relative deviation (*AARD*) for CO_2_ adsorption on of NH_2_-MIL-53(Al), DES1/NH_2_-MIL-53(Al), DES2/NH_2_-MIL-53(Al), and DES3/NH_2_-MIL-53(Al) at different temperatures (*T*).

DES/NH_2_-MIL-53	*T/*K	*H*/bar	$Q_{m}$/${\text{mg}}_{{{\text{CO}}_{{2}} }} .{\text{g}}_{{{\text{AAILs/HKUST}} - {1}}}^{{ - {1}}}$	*c/*${\text{bar}}^{ - 1}$	*R*^*2*^	^a^*AARD%*
NH_2_-MIL-53(Al)	288.15		62.521	0.306	0.9995	0.93
	293.15		60.028	0.238	0.9989	1.77
	298.15		49.646	0.261	0.9998	1.28
	303.15		40.199	0.263	0.9996	1.08
	308.15		95.485	0.046	0.9998	1.00
DES1/NH_2_-MIL-53(Al)	288.15	0.086	42.430	0.691	0.9993	1.42
	293.15	0.102	30.660	0.939	0.9999	1.33
	298.15	0.125	28.830	0.711	0.9993	1.57
	303.15	0.159	19.950	0.855	0.9998	1.10
	308.15	0.168	8.320	0.679	0.9998	0.35
DES2/NH_2_-MIL-53(Al)	288.15	0.439	48.480	0.390	0.9992	1.57
	293.15	0.379	39.670	0.364	0.9997	2.15
	298.15	0.338	23.535	0.567	0.9998	1.24
	303.15	0.274	15.857	0.364	0.9985	2.73
	308.15	0.268	3.515	0.980	0.9997	1.08
DES3/NH_2_-MIL-53(Al)	288.15	0.155	27.840	0.661	0.9983	2.44
	293.15	0.159	21.310	0.742	0.9995	1.47
	298.15	0.156	10.210	0.856	0.9998	1.00
	303.15	0.177	7.890	0.503	0.9991	1.42
	308.15	0.179	1.510	0.433	0.9984	2.17

Standard uncertainty is u(*T*) = 0.05 K.

^a^$$AARD\% = \frac{100}{n}\sum {\left| {\frac{{Q_{{CO_{2} }}^{cal} - Q_{{CO_{2} }}^{\exp } }}{{Q_{{CO_{2} }}^{\exp } }}} \right|}$$.

**Table 6:** Henry’s law constant (*H)*, $q_{m}$ and *c* are also parameters of the Redlich–Peterson isotherm model, the correlation coefficient (*R*^2^) and absolute average relative deviation (*AARD*) for N_2_ adsorption on of NH_2_-MIL-53(Al), DES1/NH_2_-MIL-53(Al), DES2/NH_2_-MIL-53(Al), and DES3/NH_2_-MIL-53(Al) at different temperatures (*T*).

DES/NH_2_-MIL-53	*T/*K	*H*/bar	$q_{m}$/${\text{mg}}_{{{\text{CO}}_{{2}} }} .{\text{g}}_{{{\text{AAILs/HKUST}} - {1}}}^{{ - {1}}}$	*c/*${\text{bar}}^{ - 1}$	*R*^*2*^	^a^*AARD%*
NH_2_-MIL-53(Al)	288.15		74.446	0.054	0.9990	1.36
	293.15		37.504	0.106	0.9998	0.76
	298.15		25.153	0.171	0.9996	1.28
	303.15		27.253	0.105	0.9991	2.29
	308.15		8.046	0.480	0.9994	1.14
DES1/NH_2_-MIL-53(Al)	288.15	0.188	11.050	0.201	0.9987	2.08
	293.15	0.286	69.180	0.013	0.9994	1.17
	298.15	0.286	29.280	0.566	0.9988	1.93
	303.15	0.395	1.250	0.251	0.9990	1.84
	308.15	0.521	0.522	0.691	0.9993	1.91
DES2/NH_2_-MIL-53(Al)	288.15	0.313	3.614	0.340	0.9992	0.65
	293.15	0.354	1.413	0.543	0.9986	1.52
	298.15	0.396	0.796	0.790	0.9988	1.57
	303.15	0.581	2.828	0.941	0.9974	1.80
	308.15	1.005	2.534	0.408	0.9988	0.89
DES3/NH_2_-MIL-53(Al)	288.15	0.240	0.734	0.779	0.9994	1.18
	293.15	0.274	0.263	0.279	0.9993	1.25
	298.15	0.327	1.347	0.956	0.9985	2.36
	303.15	0.465	1.564	0.552	0.9990	1.41
	308.15	0.554	0.966	0.726	0.9991	1.37

Standard uncertainty is u(*T*) = 0.05 K.

^a^$$AARD\% = \frac{100}{n}\sum {\left| {\frac{{Q_{{CO_{2} }}^{cal} - Q_{{CO_{2} }}^{\exp } }}{{Q_{{CO_{2} }}^{\exp } }}} \right|}$$.

### Enthalpy of adsorption

**Figure 10:**
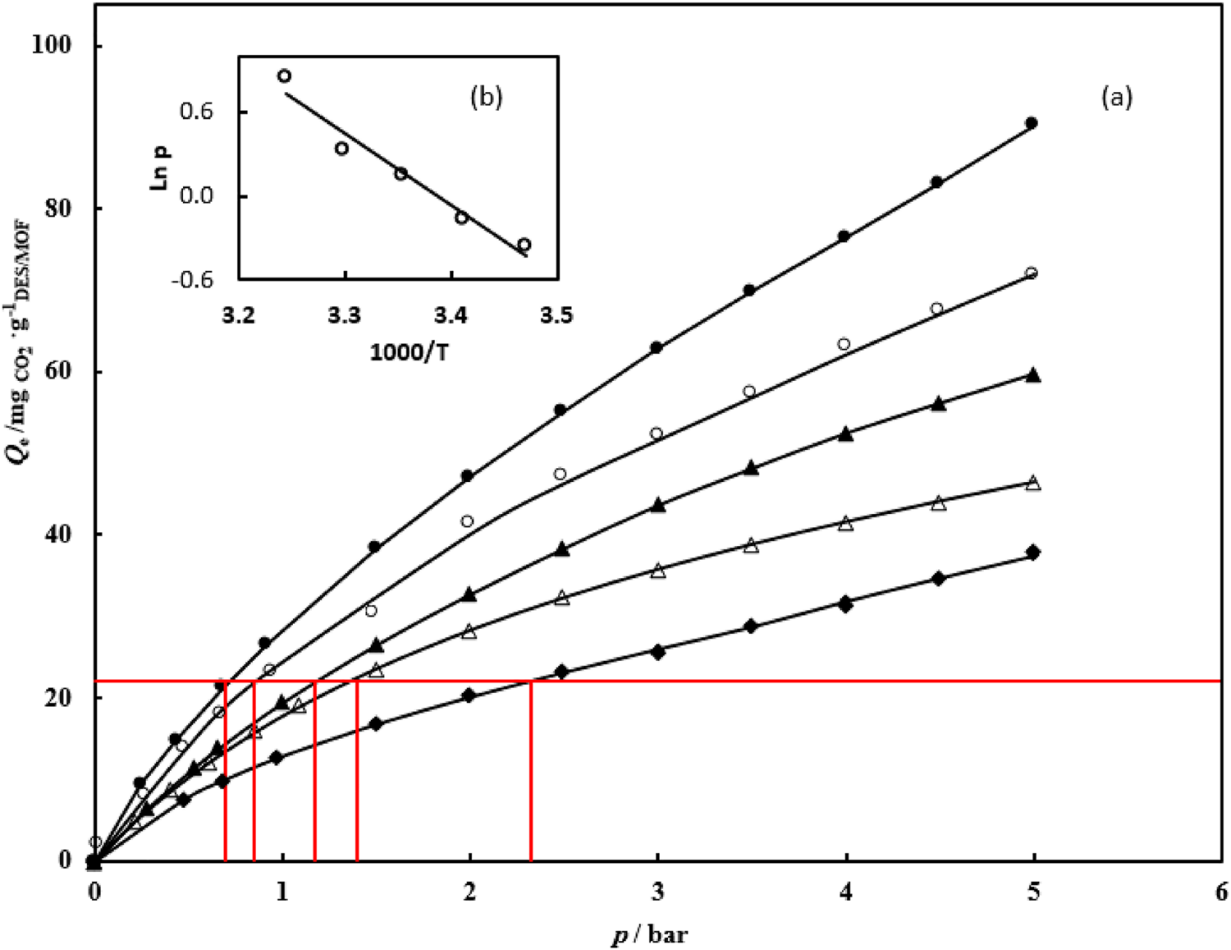
Evaluation isosteric heat of CO_2_ adsorption on DES1/NH_2_-MIL-53(Al). (**a**) Selection of isosteric pressure at different temperatures: (●) 288.15 K, (○) 293.15 K, (▲) 298.15 (Δ) 303.15 K, (♦) 308.15 K. (**b**) Plot of ln(*p*) versus 1/*T*.

In order to evaluate the heat of adsorption, the adsorption experiments were carried out in the 288.15–308.15 K range temperature. The isotherms of the studied systems indicate that the adsorption isotherm are dependent to temperature while Mahdipoor et al.^44^ indicate that the adsorption isotherm of MIL-101(Fe)–NH_2_ is independent on temperature. Hence, the value of adsorption heat is assumed to equal the activation energy for primary alkanolamines. The molar enthalpy of absorption is a measure of interaction strength between the adsorbate molecule and the adsorbent surface identified isosteric heat of adsorption. The molar enthalpy of absorption is evaluated by calculated the gas adsorption at different temperatures^45,46^. Isosteric heat has calculated by differentiate an adsorption isotherm at a constant adsorbate loading which like as the Clausius–Clapeyron equation^47,48^:

4 $$\Delta {\rm H}_{S} = R\left( {\frac{\partial \ln p}{{\partial \left( {{1 \mathord{\left/ {\vphantom {1 T}} \right. \kern-0pt} T}} \right)}}} \right)_{q} = - RT^{2} \left( {\frac{\partial \ln p}{{\partial T}}} \right)_{q}$$

where *T* and* R*, are temperature and universal gas constant, respectively. The effect of temperature on the CO_2_ adsorption in DES1/NH_2_-MIL-53(Al) sorbents is illustrated in Fig. 9. According to Fig. 10, the isosteric heat for NH_2_-MIL-53(Al) and DESs/NH_2_-MIL-53(Al) is obtained from plots ln(*p*) vs. 1/*T*. The value of isosteric heat for NH_2_-MIL-53(Al) and DESs/NH_2_-MIL-53(Al) are listed in Table 7. The calculated data for systems in this study indicate that the adsorption isotherm is dependent to temperature.

**Table 7:** The molar enthalpy of absorption for NH_2_-MIL-53 and DESs /NH_2_-MIL-53.

DES /NH_2_-MIL-53	$\Delta {\rm H}$/kJ mol^−1^	
	CO_2_	N_2_
NH_2_-MIL-53(Al)	− 38.99 ± 0.90	− 32.65 ± 0.65
DES1/NH_2_-MIL-53(Al)	− 35.50 ± 0.92	− 38.93 ± 0.15
DES2/NH_2_-MIL-53(Al)	− 40.78 ± 0.23	− 29.46 ± 0.35
DES3/NH_2_-MIL-53(Al)	− 38.52 ± 0.48	− 33.19 ± 0.90

### Regeneration efficiency of DES/NH_2_-MIL-53(Al)

**Figure 11:**
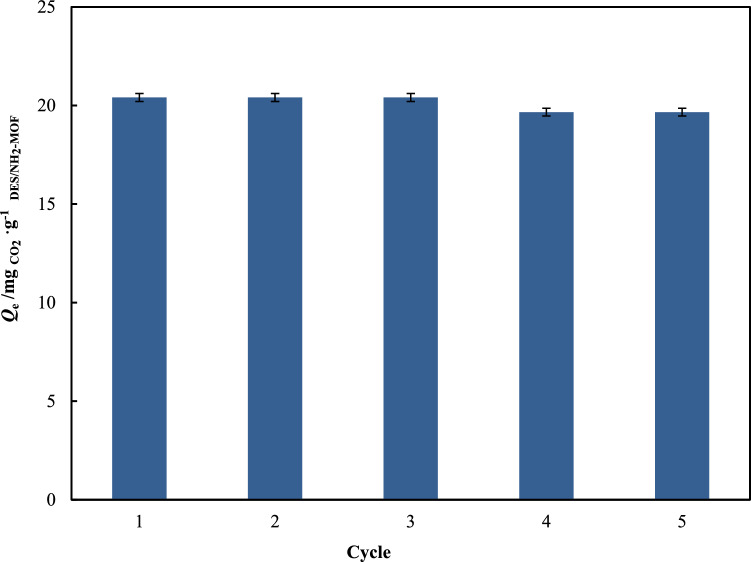
The CO_2_ absorption capacity of DES1/NH_2_-MIL-53(Al) at *p* = 1.000 bar and *T* = 298.15 K in five adsorption/desorption cycles.

Regeneration efficiency was used to evaluating adsorption/desorption performance of DES1/NH_2_-MIL-53(Al) adsorbent. Five cycles of adsorption/desorption test in DES1/NH_2_-MIL-53(Al) were tested to evaluation reuse capacity. The CO_2_ adsorption capacity in five cycles of CO_2_ adsorption/desorption test are illustrated in Fig. 11. In the adsorption/desorption test, CO_2_ adsorption has tested at 298.15 K and 1 bar and desorption test was done in vacuum condition at 298.15 K for 90 min. The values of CO_2_ adsorption are obtained as 20.408, 20.408, 20.407, 19.665 and 19.665 in five consecutive cycles of adsorption/desorption. The CO_2_ adsorption capacity reduction in the DES1/NH_2_-MIL-53(Al) compared to the fresh sample was estimated at about 4% after 5 cycles. This results confirms that the DESs/NH_2_-MIL-53(Al) is stable and reusable under the practical condition of regeneration.

## Conclusions

Deep eutectic solvents contain choline chloride in conjunction with different amines were impregnated in amino functionalized NH_2_-MIL-53(Al) to improve the separation of CO_2_/N_2_. FTIR, SEM, EDX, and N_2_-sorption analysis confirmed the impregnation of DES on porous MOF. The adsorption isotherms and separation tests of CO_2_/N_2_ revealed that DES1/NH_2_-MIL-53(Al) exhibited a better performance. The obtained results indicate that in addition to physical adsorption of CO_2_ by DES/NH_2_-MIL-53(Al), CO_2_ chemisorption by NH_2_ functional group in the sorbent structure has also a notable effect on the adsorption mechanism. The DES1/NH_2_-MIL-53(Al), can be employed repeatedly without losing separation performance and could increase the CO_2_ uptake capacity twofold which introduce a novel category of highly porous adsorbents for the efficient adsorption of different compounds.

## Data Availability

The datasets used and/or analyzed during the current study available from the corresponding author on reasonable request.
